# 24-Epibrassinolide, a Phytosterol from the Brassinosteroid Family, Protects Dopaminergic Cells against MPP^+^-Induced Oxidative Stress and Apoptosis

**DOI:** 10.1155/2011/392859

**Published:** 2011-06-03

**Authors:** Julie Carange, Fanny Longpré, Benoit Daoust, Maria-Grazia Martinoli

**Affiliations:** ^1^Department of Biochemistry, Neurosciences Research Group, Université du Québec à Trois-Rivières, Trois-Rivières, QC, Canada G9A 5H7; ^2^Neuroscience Research Unit, Centre de recherche, Université Laval, Ste-Foy, QC, Canada G1V 4G2

## Abstract

Oxidative stress and apoptosis are frequently cited to explain neuronal cell damage in various neurodegenerative disorders, such as Parkinson' s disease. Brassinosteroids (BRs) are phytosterols recognized to promote stress tolerance of vegetables via modulation of the antioxidative enzyme cascade. However, their antioxidative effects on mammalian neuronal cells have never been examined so far. We analyzed the ability of 24-epibrassinolide (24-Epi), a natural BR, to protect neuronal PC12 cells from 1-methyl-4-phenylpyridinium- (MPP^+^-) induced oxidative stress and consequent apoptosis in dopaminergic neurons. Our results demonstrate that 24-Epi reduces the levels of intracellular reactive oxygen species and modulates superoxide dismutase, catalase, and glutathione peroxidase activities. Finally, we determined that the antioxidative properties of 24-Epi lead to the inhibition of MPP^+^-induced apoptosis by reducing DNA fragmentation as well as the Bax/Bcl-2 protein ratio and cleaved caspase-3. This is the first time that the potent antioxidant and neuroprotective role of 24-Epi has been shown in a mammalian neuronal cell line.

## 1. Introduction

Parkinson's disease (PD) is characterized by the selective degeneration of nigrostriatal dopaminergic (DAergic) neurons, resulting in dopamine (DA) depletion [1]. While the etiology of PD is not completely clear, several pathology studies have demonstrated that, in* postmortem* samples of *substantia nigra pars compacta *(SNpc*), *DAergic neurons exhibit markers of oxidative stress, such as lipid peroxidation, DNA oxidative damage, and carbonyl modifications of soluble proteins [2, 3]. The PD brain is also characterized by oxidative damage and functionally impaired and misassembled mitochondrial complex I, which affirm the involvement of oxidative stress in the pathophysiology of PD [4]. Indeed, recent data have shown that Mn-dependent superoxide dismutase (SOD) level and activity are increased in PD brains [3, 5]. 

Further evidence implicating oxidative stress in PD comes from studies with the neurotoxin 1-methyl-4-phenyl-1,2,3,6-tetrahydropyridine (MPTP), which has been widely used as a DAergic neurotoxin because it causes a severe PD-like syndrome in humans as well as in monkeys and mice [6]. Its administration to C57BL mice leads to a decline of striatal DA and tyrosine hydroxylase (TH) levels in the SNpc [7, 8] as well as the death of DAergic neurons [1]. MPTP crosses the blood-brain barrier and is converted into its toxic metabolite 1-methyl-4-phenylpyridinium (MPP^+^) in astrocytes by the enzyme monoamine oxidase B. MPP^+^ is then selectively taken up by DAergic neurons via the high-affinity DA transporter (DAT) and is actively transported into mitochondria where it interferes with mitochondrial respiration through complex I inhibition [9–11], elevating reactive oxygen species (ROS) levels and increasing SOD, catalase (CAT), and glutathione peroxidase (GPx) activities in MPTP-treated mice and MPP^+^-treated neuronal cells [12–14]. Also, MPTP and its active metabolite MPP^+^ induce TH nitration, that is associated with the degeneration of DAergic neurons [15]. On the other hand, H_2_O_2_, O_2_^•−^ and ^•^OH have been detected after complex I inhibition with MPP^+^ [16]. In addition, the decrease of complex I and subsequent oxidative stress evoked by MPP^+^ administration elicit neuronal cell death by apoptosis [17, 18]. 

Brassinosteroids (BRs) are steroidal plant growth regulators found in several vegetables, including *Vicia faba* (broad bean) seed and pollen [19–21]. BRs, considered to be a new group of plant hormones, are currently being studied intensively to understand their role in plant metabolism [22]. Their main physiological effects in plants include the regulation of hormonal balance, activation of protein and nucleic acid synthesis, enzyme activity, growth promotion, increased size and quantity of fruits, and, most interestingly, augmented resistance to unfavorable environmental factors, stress, and disease (for review see [23]). It has also been demonstrated that the exogenous application of natural BRs to other vegetables has a specific antioxidative effect. The natural BR 24-epibrassinolide (24-Epi) occurring in *Vicia faba *[20] increases the enzymatic antioxidant activities of SOD, CAT, and GPx in *Lycopersicon esculentum *(tomato) leaves and *Brassica juncea* L. (Indian mustard) plants [24, 25]. 24-Epi also reduces lipid peroxidation in *Oryza sativa* L. (rice) and Indian mustard plants [25, 26].

The antioxidative properties of BRs, clearly apparent in vegetables, strongly suggest that these compounds exert an antioxidant and neuroprotective role in mammals by curbing apoptosis, as reported recently for other natural molecules (for review see [13, 27–34]). Indeed, in mammals, the effects of BRs are just starting to be elucidated. BRs are known to exert anticancer and antiproliferative activities on human cell lines [35–38]. Antiviral activity has also been identified in natural BRs and synthetic analogs [39–41].

Moreover, beans from *Vicia faba* also contain L-3,4-dihydroxyphenylalanine (L-dopa) [42, 43], the amino acid precursor of DA, which is nowadays the most effective symptomatic treatment of PD [44]. Clinical reports indicate that the consumption of *Vicia faba* has a beneficial outcome in PD patients [45, 46]. However, L-dopa concentrations in *Vicia faba* are not sufficient to explain the magnitude of the responses observed in PD patients and raise the possibility that other compounds from *Vicia faba*, such as BRs, may complement the L-dopa effect by their antioxidative activities. 

The aim of our study was to examine, in detail, the influence of 24-Epi, a BR present in *Vicia faba*, on MPP^+^-induced oxidative stress in a well-known model of PD, nerve growth factor- (NGF-) differentiated PC12 cells [47, 48]. We showed that 24-Epi reduces apoptotic cellular death as well as protein markers of apoptosis, modulates SOD, CAT, and GPx activities, and decreases intracellular ROS concentrations. Overall, our findings clearly demonstrate that 24-Epi is a new, efficient, protective molecule against MPP^+^-induced oxidative stress and might thus be regarded as a novel agent in complementary and/or preventive therapies of neurodegenerative diseases.

## 2. Materials and Methods

All reagents were purchased from Sigma (St. Louis, MO) unless stated otherwise.

### 2.1. Cell Culture and Treatments

**Figure 1:**
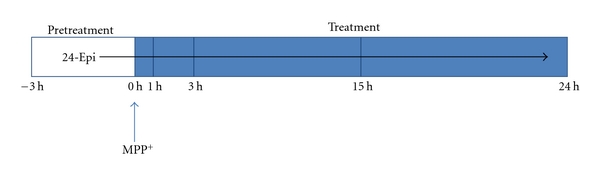
Experimental design to examine the effect of 24-Epi on MPP^+^-induced cellular death and oxidative stress. Cells were pretreated with 24-Epi (10^ −9^ M) or vehicle 3 h before MPP^+^ administration. Neuronal cells were harvested at the indicated time periods after each experimental methodology described in Section 2.

**Figure 2:**
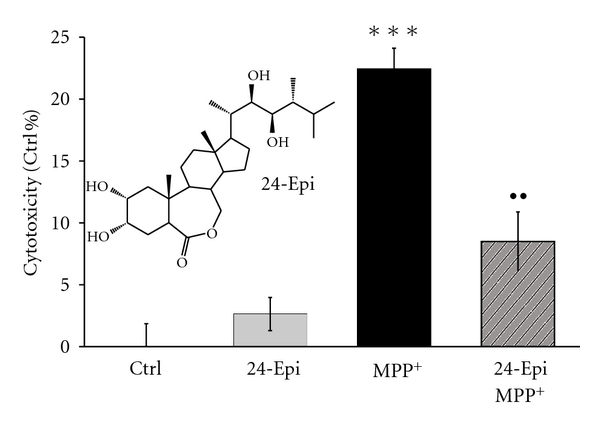
Effect of 24-Epi against MPP^+^-induced cytoxicity in neuronal PC12 cells, as measured by colorimetric assay based on LDH activity. Neuronal cells were pretreated with 24-Epi (10^−9^ M) or vehicle for 3 h and then exposed to MPP^+^ (5 mM) or not for 24 h. The absorbance value obtained for the untreated control was subtracted from all other values, as described in Section 2. Data are expressed as percentages of values of untreated control cells and are means ± S.E.M. *n* = 3. ***P < .001 versus Ctrl and ^••^ *P* < .01 versus MPP^+^.

PC12 cells, obtained from the American Type Culture Collection (Rockville, MD), were maintained in a controlled environment at 37°C and in 5% CO_2_ atmosphere. They were grown in RMPI-1640 medium supplemented with 5% fetal bovine serum (FBS), 10% horse serum, and gentamicin (50 *μ*g/mL). The culture medium was changed every 2 days and the cells were seeded at a density of 30,000 cells/cm^2^. Neuronal differentiation was induced for 4 days with 50 ng/mL NGF in RPMI-1640 medium supplemented with 1% FBS. To examine the effects of 24-Epi on MPP^+^-induced cellular death and oxidative stress, neuronal PC12 cells were pretreated with 24-Epi (10^−9^ M) or vehicle (culture medium) for 3 h and then exposed to MPP^+^ 5 mM for 1, 3, 15, or 24 h [49, 50] (Figure 1). In apoptosis experiments, we used 500 *μ*M of MPP^+^ for 24 h, as reported elsewhere [49–51]. After kinetics and dose-response studies [49, 50], the final concentration of 10^−9^ M 24-Epi (see Figure 2 for chemical structure) was chosen as the lowest dose capable of rescuing cells from MPP^+^-induced cellular death (data not included). All experiments were performed in phenol red-free medium and charcoal-stripped serum to remove steroids from the medium.

### 2.2. Cytotoxicity

Cytotoxicity was evaluated by colorimetric assay based on the measurement of lactate dehydrogenase (LDH) activity released from damaged cells into the supernatant [52]. LDH, a stable cytoplasmic enzyme present in all cells, is rapidly released into the cell culture supernatant upon plasma membrane injury. The amount of enzyme activity detected in the culture supernatant correlates with the proportion of lysed cells [53, 54]. 

NGF-differentiated PC12 cells were grown and treated in collagen-coated 96-well plates. Then, 100 *μ*L of LDH substrate mixture was added to 50 *μ*L of cell-free supernatant, as described elsewhere [49]. Absorbance was measured at a wavelength of 490 nm in a microplate reader (Thermolab System, Franklin, MA). Total cellular LDH was determined by lysing the cells with 1% Triton X-100 (high control); the assay medium served as a low control and was subtracted from all absorbance measurements:

(1) $$\begin{array}{cc} & \text{Cytotoxicity}\;\left(\text{\%}\right)\\ & \;=\frac{\left(\text{Experimental}\;\text{value}-\text{Low}\;\text{control}\right)\;}{\left(\text{High}\;\text{control}-\text{Low}\;\text{control}\right)}\times 100. \end{array}$$

### 2.3. ROS Detection

The antioxidative effect of 24-Epi against MPP^+^-induced ROS was evaluated by dihydrorhodamine (DHR) 123 assay and MitoSOX Red (Invitrogen, Toronto, ON, Canada), according to a previously-described method [13, 55]. Briefly, to detect OH^•^, NO_2_^•^, CO_3_^•−^, H_2_O_2_, HOCl, and ONOO^−^ by DHR [56–59], NGF-differentiated PC12 cells were grown and treated on collagen-coated slides in 24-well plates. A stock solution of DHR was prepared in dimethylsulfoxide under nitrogen, to a concentration of 10 mM and stored at −80°C. After 3-hour pretreatment with 24-Epi, MPP^+^ was added for 3-hour or 24-hour treatment (Figure 1). Then, the neuronal PC12 cells were quickly washed with PBS 0.1 M and exposed to 250 *μ*L of DHR at 37°C for 20 min. Slides with live cells were immediately examined under a Leitz Orthoplan fluorescence microscope (Leica, Wetzlar, Germany) and photographed with a Qimaging camera (Nikon, Mississauga, ON, Canada). Fluorescence intensity was measured by NIS Elements 2.2 software (Nikon).

Then, the antioxidative effect of 24-Epi against MPP^+^-induced O_2_^•−^ was evaluated with MitoSOX Red, according to the manufacturer's protocol. To show the selectivity of MitoSOX Red, 80 *μ*M of* N,N-*diethyldithiocarbamate (DDC), an inhibitor of SOD, was used as a positive control. After 3-hour pretreatment with 24-Epi, MPP^+^ was added for 3-hour or 24-hour treatment (Figure 1). PC12 cells were washed with Hanks' buffered salt solution and incubated for 10 min with MitoSOX Red 5 *μ*M solution at 37°C. They were then counterstained in blue with Hoechst 33342 for 10 min at 37°C, fixed for 15 min in 4% paraformaldehyde at 37°C, and finally mounted with ProLong Antifade kits (Invitrogen). The slides were examined under a Leitz Orthoplan fluorescence microscope (Leica) and photographed with a Qimaging camera (Nikon). Fluorescence intensity was measured by NIS Elements 2.2 software (Nikon). 

### 2.4. SOD, CAT, and GPx Activities

NGF-differentiated PC12 cells were grown and treated in collagen-coated 6-well plates. After 3-hour pretreatment with 24-Epi, MPP^+^ was added for 1, 3, 15, or 24 h (Figure 1). Neuronal cells were harvested mechanically and collected by centrifugation at 2,000 g for 10 min at 4°C. For SOD and GPx activities, the pellets were homogenized in 1 mL of cold PBS and centrifuged at 2,000 g for 10 min at 4°C. The supernatants were discarded and the freeze-thaw method was performed to break the cells (−20°C for 20 min, followed by a 37°C bath for 10 min, repeated twice). The pellets were homogenized in cold PBS and centrifuged at 10,000 g for 15 min at 4°C. Finally, the supernatant was analyzed according to the manufacturer's protocol (SOD Assay Kit-WST, Dojindo Molecular Technologies, Gaithersburg, MD; GPx Assay Kit, Cayman Chemical, Ann Arbor, MI). The reaction was monitored at 450 nm for SOD activity and 340 nm for GPx activity in a microplate reader (Thermolab System). For CAT activity, the pellets were homogenized in 1 mL of cold buffer (50 mM potassium phosphate, pH 7.0, containing 1 mM EDTA) and sonicated (3 times, 5 s). The samples were centrifuged at 10,000 g for 15 min at 4°C, and the supernatant was assayed according to the manufacturer's protocol (Catalase Assay Kit, Cayman Chemical). The reaction was monitored at 540 nm in a microplate reader (Thermolab System).

### 2.5. Apoptosis Detection

Apoptotic neuronal cells were detected by both terminal deoxynucleotidyl transferase dUTP nick end labeling (TUNEL, Roche Diagnostics, Laval, QC, Canada) and activated caspase-3 immunofluorescence. Neuronal PC12 cells were grown and treated on collagen-coated circular glass coverslips in 24-well plates (Fischer Scientific, Ottawa, ON, Canada). After 3-hour pretreatment with 24-Epi, MPP^+^ (500 mM) was added for 24 h (Figure 1). The cells were fixed for 15 min in 4% paraformaldehyde at 37°C, washed, and incubated in a blocking and permeabilizing solution (containing 1% BSA, 0.18% fish skin gelatin, 0.1% Triton-X, and 0.02% sodium azide) for 30 min at room temperature (RT). They were then incubated with anticleaved caspase-3 antibody (New England Biolabs, Pickering, ON, Canada) diluted 1 : 500, for 2 h at RT, followed by 90-minute incubation with a Cy3-conjugated secondary antibody (Medicorp, Montreal, QC, Canada) diluted 1 : 500 for 1 h at 4°C. The coverslips were then transferred to the TUNEL reaction mixture in a humidified atmosphere at 37°C. The cells were rinsed with PBS, nuclei were counterstained in blue with DAPI for 10 min at 37°C, and mounted with ProLong Antifade kits (Invitrogen). Images were acquired with a Leitz Orthoplan fluorescence microscope (Leica) and photographed with a Qimaging camera (Nikon). Neuronal cells were considered to be apoptotic when they were positive for cleaved caspase-3 and their nuclei were stained by TUNEL. Z-DEVD-FMK (Bachem, Torrance, CA), a cell-permeable caspase-3 inhibitor, was used on specific wells of neuronal PC12 as internal control for caspase-3 activation (Figure 7). The number of apoptotic neuronal cells among 300 randomly chosen neuronal cells was counted on 10 different optical fields from 3 slides per group, as already reported [51], with NIS Elements 2.2 software (Nikon).

In addition, DNA fragmentation was assessed with single-stranded DNA (ssDNA) apoptosis ELISA kits (Chemicon International, Temecula, CA) according to the manufacturer's instructions, to quantify ssDNA present in apoptotic cells. This procedure is based on the ability of a monoclonal antibody to detect ssDNA, which occurs in apoptotic cells but not in necrotic cells or in cells with DNA breaks in the absence of apoptosis. The assay involves the binding of cells to 96-well plates and treatment of the attached cells with formamide which selectively denaturates DNA in apoptotic cells. A mixture of anti-ssDNA monoclonal antibody and peroxidase-conjugated secondary antibody served to specifically identify apoptotic cells. The reaction was stopped and ssDNA fragmentation was quantified by measuring absorbance at 405 nm in a microplate reader (Thermolab System). The amount of ssDNA was calculated with reference to control conditions. To confirm assay specificity, positive (ssDNA fragment) and negative (S1 nuclease-treated cells) controls were included.

### 2.6. Electrophoresis and Immunoblotting Analysis

NGF-differentiated PC12 cells were grown and treated in collagen-coated 6-well plates. After 3-hour pretreatment with 24-Epi, MPP^+^ was added for 24 h (Figure 1). Total cellular proteins were extracted with Nuclear Extraction Kits (Active Motif, Carlsbad, CA), diluted in 50 *μ*L of lysis solution, and their concentrations quantified by protein assay (BCA Protein Assay Kit; Pierce, Rockford, IL). Equal amounts of proteins were loaded onto 12% polyacrylamide gel-sodium dodecyl sulfate. After electrophoretic separation (180 V, 1 h), the polyacrylamide gels were transferred onto nylon PVDF membranes (0.22-*μ*m pore size, BioRad, Hercules, CA) at 60 V for 2 h. The membranes were blocked with 5% nonfat powder milk for 1 h at RT. Immunoblotting was probed overnight at 4°C with the primary antibody. Rabbit anti-Bax antibody (Delta Biolabs, Gilroy, CA) was diluted 1 : 1,000, and rabbit anti-Bcl2 antibody (Santa Cruz Biotechnology, Santa Cruz, CA), 1 : 50. The membranes were washed the following day, and antirabbit horse-radish peroxidase-conjugated secondary antibody diluted 1 : 10,000 was added for 2 h at RT. Immunopositive signals were visualized by enhanced chemiluminescence with the AlphaEase FC imaging system (Alpha Innotech, San Leandro, CA) and analyzed with AlphaEase FC software (Alpha Innotech).

### 2.7. Statistical Analysis

Significant differences between groups were determined by 1-way ANOVA, followed by Tukey's post hoc analysis with the GraphPad Instat program, version 3.06, for Windows (San Diego, CA, http://www.graphpad.com/welcome.htm). All data, analyzed at the 95% confidence interval, are expressed as means ± S.E.M. from 3 independent experiments. Asterisks indicate statistical differences between the treatment and respective control condition (***P < .001, **P < .01, and *P < .05), full circles show statistical differences between the treatment and MPP^+^ condition (^•••^ *P* < .001, ^••^ *P* < .01, and ^•^ *P* < .05), and diamonds denote statistical differences between the treatment and DDC condition (^*♦♦♦*^ *P* < .001, ^*♦♦*^ *P* < .01, and ^*♦*^ *P* < .05). 

## 3. Results

### 3.1. 24-Epi Reduces Cytotoxicity and ROS Production Induced by MPP^+^

The ability of 24-Epi to reverse MPP^+^-induced cytotoxicity was investigated by LDH colorimetric assay [13, 51]. Cytotoxicity measurements revealed significant cell death in neuronal PC12 cells after exposure to MPP^+^ for 24 h (Figure 2, MPP^+^). Specifically, MPP^+^ induced 22% cell death whereas 24-Epi, when used alone, did not cause any cellular mortality (Figure 2, 24-Epi). Three-hour pretreatment with 24-Epi before the induction of oxidative stress significantly decreased MPP^+^-induced cytotoxicity. Specifically, 24-Epi partially protected neuronal PC12 cells against MPP^+^ toxicity by decreasing cellular death by 60% (Figure 2, 24-Epi + MPP^+^).

**Figure 3:**
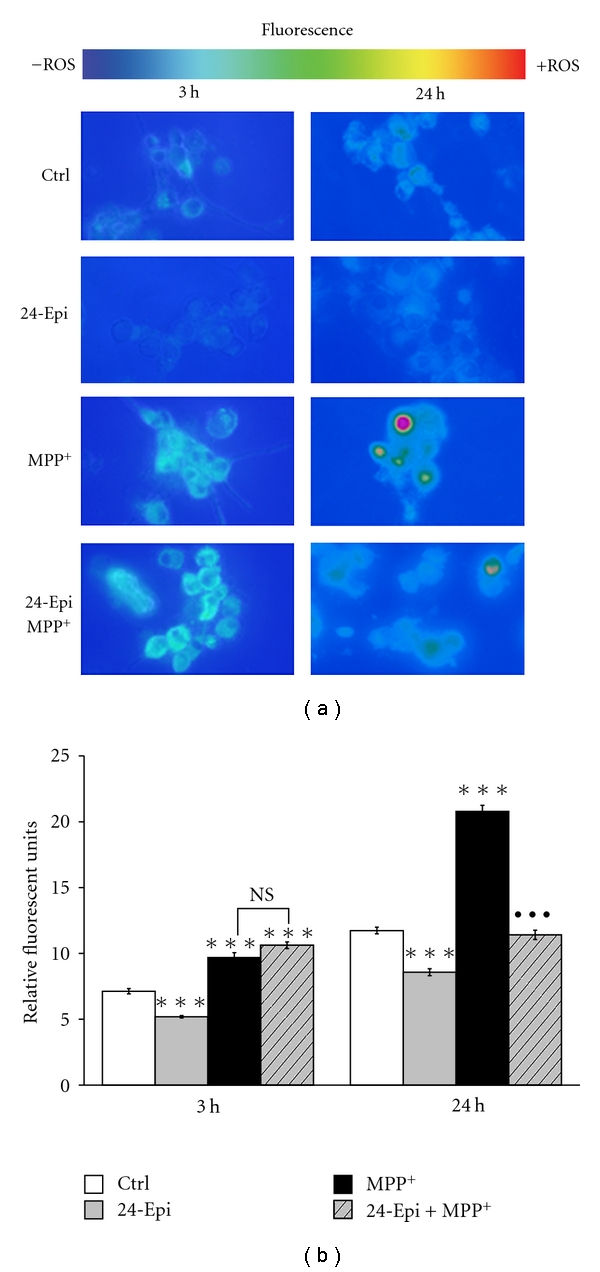
Rhodamine detection of ROS in neuronal PC12 cells after 3-hour and 24-hour treatment. Nonfluorescent DHR is converted to fluorescent rhodamine in the presence of several free radicals (OH^•^, NO_2_^•^, CO_3_^•−^, H_2_O_2_, HOCl, ONOO^−^). (a) Fluorescence microphotographs after 3-hour or 24-hour treatment. Cells were pretreated with 24-Epi or vehicle (Ctrl) for 3 h. Then, they were treated or not with MPP^+^ for 3 h or 24 h. A marked signal of fluorescent rhodamine is evident in neuronal cells treated with MPP^+^ but not in those exposed to the vehicle (Ctrl) or 24-Epi alone (24-Epi). A striking reduction of fluorescence is evident in cells pretreated with 24-Epi and then treated with MPP^+^ (24-Epi + MPP^+^) compared to cells pretreated with vehicle and treated with MPP^+^ (MPP^+^). (b) Semiquantitative image analysis of fluorescent cells after 3-hour and 24-hour treatment. Data are expressed as relative fluorescent units and are means ± S.E.M. Magnification 400x. *n* = 3. ***P < .001 versus Ctrl and ^•••^ *P* < .001 versus MPP^+^.

Figure 3 depicts the preventive effect of 24-Epi against MPP^+^-induced oxidative stress measured by DHR assay. DHR, a nonfluorescent dye, is oxidized to highly fluorescent rhodamine in the presence of several free radicals (OH^•^, NO_2_^•^, CO_3_^•−^, H_2_O_2_, HOCl, and ONOO^−^) [56–59].  Figure 3(a) illustrates low levels of rhodamine fluorescence in control neuronal PC12 cells, treated only with vehicle (Figure 3(a), Ctrl), as well as in cells exposed to 24-Epi alone (Figure 3(a), 24-Epi). In contrast, a marked signal was detected in neuronal cells treated with MPP^+^ for 24 h (Figure 3(a), MPP^+^). Pretreatment with 24-Epi prior to MPP^+^ revealed a dampened signal in comparison to MPP^+^ alone (Figure 3(a), 24-Epi + MPP^+^), indicating a preventive role of 24-Epi in MPP^+^-induced ROS production. Semiquantitative image analysis (Figure 3(b)) disclosed high levels of fluorescent rhodamine only in neuronal PC12 cells treated with MPP^+^ for 24 h and a statistically significant reduction (*P* < .001) when they were preincubated with 24-Epi prior to the induction of oxidative stress (Figure 3(b), 24 h). A modest increment of fluorescence was evident after 3 h of MPP^+^ administration (Figure 3(b), 3 h), indicating that longer exposure to MPP^+^ is needed to show the presence of several free radicals, such as OH^•^, NO_2_^•^, CO_3_^•−^, H_2_O_2_, HOCl, and ONOO^−^.

**Figure 4:**
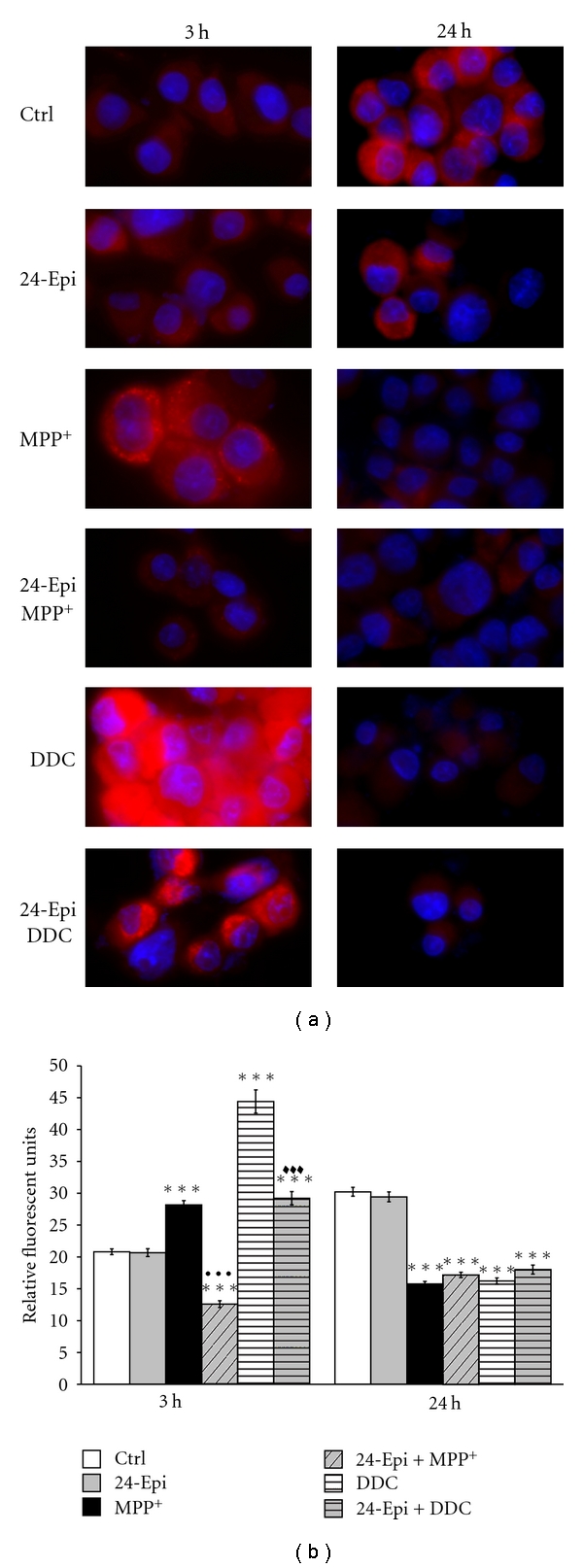
Selective detection of O_2_^•−^ by MitoSOX Red in neuronal PC12 cells. Once in the cells, this fluorogenic dye is oxidized by superoxide and exhibits red fluorescence. (a) Fluorescence microphotographs at 3-hour or 24-hour treatment. Cells were pretreated with 24-Epi or vehicle (Ctrl) for 3 h. Then, they were treated or not with MPP^+^ for 3 h or 24 h. A significant signal of MitoSOX Red is apparent in neuronal cells treated with MPP^+^. This signal is much less intense in cells exposed to vehicle (Ctrl) or 24-Epi alone (24-Epi). A marked reduction of red fluorescence is also clear in cells pretreated with 24-Epi and then treated with MPP^+^ (24-Epi + MPP^+^). DDC: cells were treated with 80 *μ*M DDC for 3 h or 24 h. 24-Epi + DDC: cells were pretreated with 10^−9^ M 24-Epi, then with 80 *μ*M DDC for 3 h or 24 h. (b) Semiquantitative image analysis of fluorescence at 3 h and 24 h. Nuclei were counterstained in blue with Hoechst 33342. Data are expressed as relative fluorescent units and are means ± S.E.M. Magnification 400x. *n* = 3. ***P < .001 versus Ctrl, ^•••^ *P* < .001 versus MPP^+^, and ^*♦♦♦*^ *P* < .001 versus DDC.

In addition, the selective detection of O_2_^•−^ by the fluorogenic dye MitoSOX Red is illustrated in Figure 4(a), in neuronal PC12 cells after 3 h of pretreatment with 24-Epi or vehicle (Ctrl), and then 3-hour or 24-hour treatment with MPP^+^. It should be noted that we performed complete kinetics analysis at 3, 15, and 24 h (Figure 1). However, we detected considerable levels of fluorescence, that is, O_2_^•−^, only at 3 h of treatment (Figure 4(a)). DDC was employed as positive control. Fluorescence pictures revealed high fluorescence intensity in MPP^+^- and DDC-treated cells after 3-hour treatment. Low levels of oxidized MitoSOX Red were detected in control neuronal PC12 cells (Figure 4(a), Ctrl) as well as in cells receiving only 24-Epi (Figure 4(a), 24-Epi). Pretreatment with 24-Epi provoked a marked reduction of the red fluorescence signal induced by MPP^+^ or DDC (Figure 4(a), 24-Epi + MPP^+^ and 24-Epi + DDC). Semiquantitative image analysis disclosed high levels of fluorescent MitoSOX Red in neuronal PC12 cells treated with MPP^+^ or DDC and a considerable reduction (*P* < .001) of fluorescence intensity when these cells were pretreated with 24-Epi prior to MPP^+^ or DDC (Figure 4(b)) for 3 h. Marginal or slightly detectable levels of fluorescence were apparent at 15 h (data not included) and at 24 h of treatment (Figure 4(b), 24 h). 

### 3.2. Effects of 24-Epi on SOD, CAT, and GPx Activities

**Figure 5:**
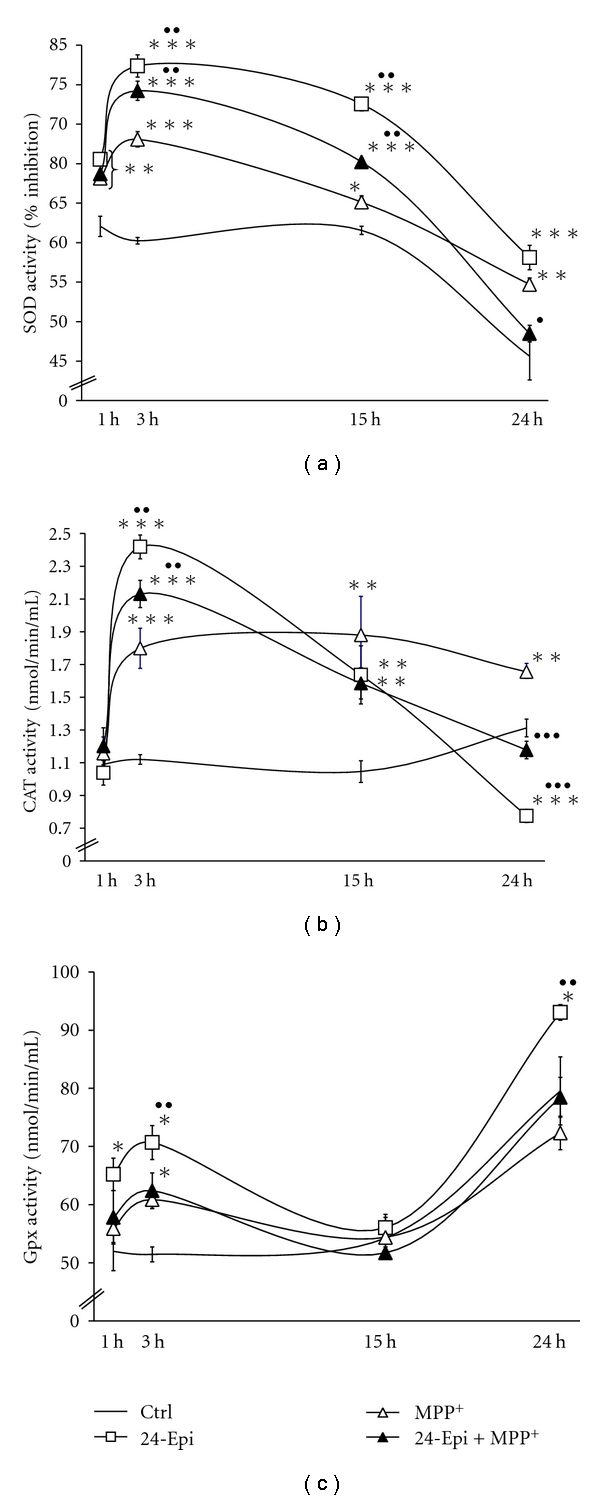
Time course changes in antioxidant enzyme activities in neuronal PC12 cells. (a) SOD activity: administration of 24-Epi alone (□) induced higher SOD activity than MPP^+^ (∆). Preincubation with 24-Epi prior to MPP^+^ (▲) also augmented SOD activity. A maximal increase was apparent after 3 h of treatment. (b) CAT activity: MPP^+^ (∆) induced a constant elevation of CAT activity until 24 h. 24-Epi (□) heightened CAT activity after 3 h of incubation with a subsequent decline in CAT activity becoming less active than the control after 24-h exposure. Pretreatment with 24-Epi before MPP^+^ (▲) increased CAT activity similarly to that observed in cells treated with 24-Epi alone. (c) GPx activity: pretreatment with 24-Epi (□) induced a maximal increment of GPx activity at 24 h while the increase with MPP^+^ (∆) and 24-Epi + MPP+ (▲) was less pronounced. Data are expressed as means ± S.E.M. *n* = 3. *P < .05, **P < .01, and ***P < .001 versus Ctrl; ^•^ *P* < .05, ^••^ *P* < .01, and ^•••^ *P* < .001 versus MPP^+^.

SOD catalyzes the dismutation of superoxide anion radical by converting it to peroxide, which can be destroyed by CAT or GPx [60, 61]. We, therefore, investigated the effect of MPP^+^, 24-Epi, and MPP^+^ + 24-Epi on SOD, CAT, and GPx activities in neuronal PC12 cells. Figures 5(a)–5(c), illustrating SOD, CAT, and GPx activities, respectively, reveal that the exposure of neuronal PC12 cells to MPP^+^ for 3 h significantly increased the levels of these 3 antioxidant enzymes, corroborating a cell stress response to MPP^+^-induced ROS production [13]. At 15 h, MPP^+^ administration decreased SOD and GPx activities (Figures 5(a) and 5(c), MPP^+^), with CAT activity remaining stable relative to 3 h activity (Figure 5(b), MPP^+^) whereas at 24 h, SOD activity was strongly reduced, CAT activity remained stable, and GPx was increased. Our results show that the administration of 24-Epi alone also induced a very significant rise of SOD, CAT, and GPx activities at 3 h, supporting an antioxidant role for this BR (Figures 5(a)–5(c), 24-Epi). Specifically, 24-Epi elicited a significant increment of SOD, CAT, and GPx activities, with a maximal increase apparent after 3 h of treatment. Afterward, at 15 h and later, SOD and CAT activities declined when exposed to 24-Epi (Figures 5(a) and 5(c), 24-Epi). On the other hand, Figure 5(c) illustrates that GPx activity declined at 15 h and then peaked after incubation with 24-Epi for 24 h. We also analyzed whether 24-Epi administration prior to MPP^+^ could modulate the activities of these antioxidant enzymes. We found that exposure to 24-Epi before the induction of MPP^+^ oxidative damage significantly increased SOD, CAT, and GPx at 3 h, to levels higher than those obtained with MPP^+^ alone but lower than those detected with 24-Epi alone. At 15 h, our results demonstrate that 24-Epi + MPP^+^ still elevated SOD activity over control values (Figure 5(a), 24-Epi + MPP^+^) while CAT activity was similar to that observed with 24-Epi alone (Figure 5(b), 24-Epi + MPP^+^), and GPx activity declined significantly. Finally, at 24 h, SOD and CAT activities decreased markedly, while GPx activity increased to control levels (Figures 5(a)–5(c), 24-Epi + MPP^+^).

### 3.3. 24-Epi Reduces MPP^+^-Induced Apoptosis

**Figure 6:**
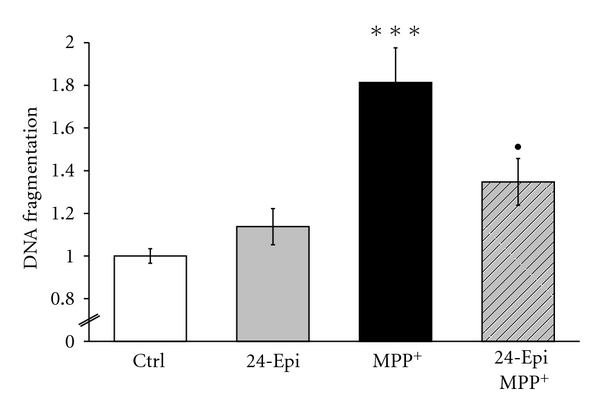
Detection of DNA fragmentation in apoptotic cells with monoclonal antibody to ssDNA. Cells were pretreated with 24-Epi or vehicle (Ctrl) for 3 h. Then, they were treated or not with MPP^+^ for 24 h. PC12 cells pretreated with vehicle and exposed to MPP^+^ for 24 h (MPP^+^) revealed a significant increase in DNA fragmentation compared to the control (vehicle). Pretreatment with 24-Epi and treatment with MPP^+^ (24-Epi + MPP^+^) significantly decreased DNA fragmentation compared to the MPP^+^ condition. The data are expressed as percentages of values in untreated control cells and are means ± S.E.M. *n* = 3. ***P < .001 versus Ctrl and ^•^ *P* < .05 versus MPP^+^.

**Figure 7:**
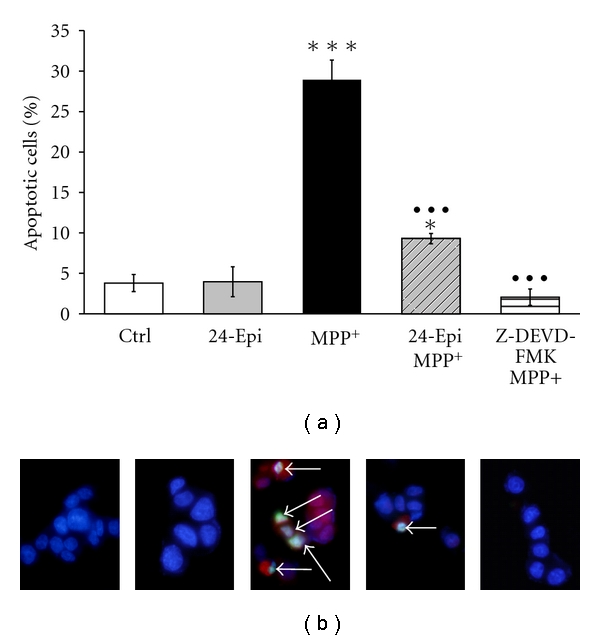
(a) Histogram showing the percentage of apoptotic cells counted on triple-stained slides (merged), as described in Materials and Methods. To illustrate that caspase-3 activation is a key step in the MPP^+^-induced apoptotic pathway, PC12 neuronal cells were pretreated with Z-DEVD-FMK, a cell-permeable caspase-3 inhibitor, followed by treatment with MPP^+^. Pretreatment with 24-Epi strongly reduced the number of apoptotic neuronal PC12 cells induced by treatment with MPP^+^ (24-Epi + MPP^+^), and pretreatment with the caspase-3 inhibitor prevented MPP^+^-induced apoptosis. The data are expressed as percentages of apoptotic cells and are means ± S.E.M. *n* = 3. *P < .05 and ***P < .001 versus Ctrl and ^•••^ *P* < .001 versus MPP^+^. (b) Immunofluorescence detection of apoptotic neuronal PC12 cells on triple-stained slides (merged), as described in Materials and Methods. Blue: neuronal PC12 nuclei were counterstained in blue with DAPI. Red: anticleaved caspase-3 antibody. Green: TUNEL. Merged: cells are considered apoptotic when positive for both TUNEL (green) and anticleaved caspase-3 antibody (red). They appear as light blue cells (arrows) because of the third superposition of dark blue DAPI. Triple-staining (merged) clearly reveals several apoptotic cells, indicated by arrows, on slides treated with MPP^+^ (MPP+) and fewer apoptotic PC12 cells (arrow) when they are pretreated with 24-Epi prior to MPP^+^ addition.

To determine whether 24-Epi protects neuronal PC12 cells from MPP^+^-induced apoptosis, we undertook DNA fragmentation measurement (Figure 6), TUNEL assay, and immunofluorescence investigation with an antibody to activated caspase-3 (Figures 7(a) and 7(b)). DNA fragmentation is a marker of late apoptosis, and exposure to 500 *μ*M MPP^+^ for 24 h resulted in its 60% increase in neuronal PC12 cells (Figure 6). Pretreatment with 24-Epi significantly (*P* < .01) prevented the MPP^+^-induced increment of DNA fragmentation, indicating a powerful role of 24-Epi in reducing apoptosis in our cell paradigm. Next, caspases are central initiators and executioners of the complex biochemical events associated with apoptotic cell death [62, 63]. As caspase-3 activation has been shown to be one of the concluding effectors of the apoptosis process [64], we investigated whether 24-Epi has the ability to prevent MPP^+^-induced caspase-3 activation (Figure 7). Immunofluorescence (Figure 7(b)) clearly illustrated the presence of simultaneous TUNEL- and caspase-3-positive cells (appearing in light blue and indicated by arrows) when MPP^+^ was administered alone. Furthermore, pretreatment of neuronal PC12 cells with a cell-permeable caspase-3 inhibitor (Z-DEVD-FMK) for 1 h prior to MPP^+^ significantly decreased MPP^+^-induced apoptosis, demonstrating that caspase-3 activation is a key factor in MPP^+^-induced apoptosis. As already depicted in Figure 6, 3-hour preincubation with 24-Epi prior to MPP^+^ revealed a considerable reduction (*P* < .001) in the number of apoptotic neuronal PC12 cells (Figure 7(b)). These results strongly suggest an antiapoptotic effect of 24-Epi and indicate that MPP^+^-induced apoptosis is associated with caspase-3 activation.

### 3.4. 24-Epi Modulates Bax/Bcl-2 Protein Expression

**Figure 8:**
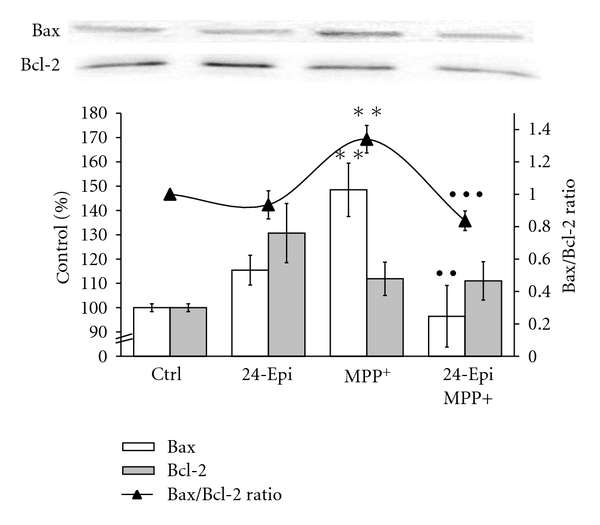
Effect of 24-Epi on the Bax/Bcl-2 ratio in neuronal PC12 cells. Cells were pretreated with 24-Epi or vehicle (Ctrl) for 3 h. Then, they were treated or not with MPP^+^for 24 h. Bax and Bcl-2 protein expression levels were quantified and the Bax/Bcl-2 ratio was determined for each treatment (-▲-). Pretreatment with 24-Epi alone did not modulate the Bax/Bcl-2 ratio (24-Epi). In contrast, the Bax/Bcl-2 ratio increased upon treatment with MPP^+^ while pretreatment with 24-Epi (24-Epi + MPP^+^) significantly prevented this increment. The data are expressed as percentages of values in untreated control cells and are means ± S.E.M. *n* = 3. **P < .01 versus Ctrl, ^••^ *P* < .01 versus MPP^+^, and ^•••^ *P* < .001 versus MPP^+^.

We also studied the modulation of protein expression of the proapoptotic gene Bax and the antiapoptotic gene Bcl-2 by 24-Epi. The ratio of proapoptotic Bax to antiapoptotic Bcl-2 (Bax/Bcl-2) has been reported to be correlated with apoptosis [51, 65]. Our results reveal that the administration of 24-Epi alone did not significantly modulate the Bax/Bcl-2 ratio (Figure 8, triangles on a continuous line). Treatment with MPP^+^ alone significantly increased the Bax/Bcl-2 ratio, indicating that MPP^+^-induced apoptosis of PC12 cells may be mediated by the mitochondrial pathway. The MPP^+^-induced increase of the Bax/Bcl-2 ratio was considerably attenuated in cells pretreated with 24-Epi (Figure 8, 24-Epi + MPP^+^) to control levels, suggesting, for the first time, that the BR 24-Epi is a strong modulator of proapoptotic and antiapoptotic gene expression.

## 4. Discussion

In this paper, we demonstrated, for the first time, that 24-Epi, a BR found in a variety of vegetables as well as in *Vicia faba,* can exert antioxidative and consequent antiapoptotic actions in mammalian neural cells. In particular, we studied PC12 cells, a known, reliable, and efficient model for the investigation of oxidative stress and neuroprotection of DA neurons [49, 66]. After NGF administration, PC12 cells differentiate into a neuronal-like phenotype that secretes high DA levels and expresses TH, DAT, neurofilaments as well as estrogen receptor-alpha and -beta (ER*α* and ER*β*) [49, 66–68].

Recent studies have reported the powerful properties of various natural polyphenols against oxidative stress in several cellular and *in vivo* paradigms of neurodegenerative disease [30–34]. Currently, many natural polyphenols are under intense investigation for their antioxidative effects and their possible use as complementary and/or preventive therapies of diseases [33, 34]. Our aim was to demonstrate that BRs, contained in a wide variety of vegetables, indeed exert antioxidative as well as neuroprotective properties in neuronal PC12 cells, a cellular model of PD [47, 48]. 

At present, phytosterols are recognized antioxidants [69], and some of them possess antioxidative properties associated with neuroprotective effects [70, 71]. Others, such as *β*-sitosterol, modulate SOD, GPx, and CAT activities [72], and the ginsenoside Rg1, a phytosterol derived from ginseng, is also reported to be antiapoptotic in neuronal PC12 cells after oxidative stress [73, 74]. However, BRs, in particular, are much less studied, even if the recent literature is pointing to their interesting potential in mammalian systems, such as antiviral [40], anticancer, and antiproliferative activities [35–37]. At present, no data on a possible antioxidative and antiapoptotic role of BRs are available in mammalian neurons *in vitro* or *in vivo*. As such, in this study, we examined, for the first time, the neuroprotective, antioxidant, and antiapoptotic consequences of low-dose 24-Epi (10^−9^ M), a common BR, against oxidative damage induced by treatments with MPP^+^, the active metabolite of MPTP, a known Parkinsonian toxin. The positive outcomes we reported in DAergic neuronal culture, using nanomolar doses of 24-Epi, on parameters of neuroprotection, oxidative metabolism, and apoptosis, are supported by the fact that BRs may be considered the plant equivalent of steroid hormones in vertebrates, sharing similar metabolic pathways [75]. Thus, BRs could easily pass through the blood-brain barrier and are likely to accumulate in the brain and serum, as demonstrated for plant sterol and stanol esters in Watanabe rabbits [76]. In particular, we established that 24-Epi can protect DA neuronal cells from MPP^+^-induced cellular death by reducing intracellular ROS production. Indeed, nonfluorescent DHR has the capacity to enter cells and, once inside them, it is oxidized by oxygen species (superoxide anion, peroxynitrite) to fluorescent rhodamine [57]. Accordingly, our results show increased rhodamine fluorescence after MPP^+^ treatment and reduced fluorescence when 24-Epi is administered to neuronal PC12 cells prior to MPP^+^. DHR has been deployed extensively to measure intracellular ROS, but it does not quantify O_2_^•−^ production. MitoSOX Red is a selective indicator of mitochondrial O_2_^•−^ production and becomes highly fluorescent when oxidized by this ROS but not by other oxidants. With MitoSOX Red, we illustrated an increase of fluorescence when MPP^+^ was administered alone for 3 h, and a substantial reduction with 24-Epi treatment given prior to MPP^+^ for 3 h, suggesting a potent scavenging role for 24-Epi. As O_2_^•−^ is a highly reactive ROS, we could barely detect its presence by MitoSOX Red at 15 or 24 h of treatment. However, at the cellular level, since antioxidant enzymes are the primary defense mechanisms of protection against ROS damage, SOD, CAT, and GPx are pivotal in preventing cellular injury and apoptosis. In our study, augmented SOD activity demonstrated that 24-Epi may enhance the ability to eliminate ROS during various oxidative stresses and may indicate a protective role in pretreatment experiments. Besides, several other natural and synthetic molecules are reported to heighten SOD activity in various cellular systems [13, 77–79]. MPP^+^ augmented SOD activity in our experiments, as described in recent literature *in vitro *[13] and *in vivo*, where MPTP increased SOD activity by generating superoxide ions [14]. This apparent contrasting result should be analyzed by comparing it with those obtained by fluorescent rhodamine and MitoSOX Red. Indeed, low ROS levels, illustrated by low rhodamine and low MitoSOX Red fluorescence (24-Epi and 24-Epi + MPP^+^, Figures 3 and 4), sustain the ability of 24-Epi to induce SOD activity, as demonstrated by our data. When MPP^+^ was administered, rhodamine and MitoSOX Red fluorescence indicated high ROS levels and, consequently, the cells responded by augmenting SOD activity, as already reported [13, 14]. Our findings clearly show that SOD activity may be induced by 2 different mechanisms, a protective mechanism (24-Epi) and a response-to-stress mechanism (MPP^+^). More importantly, pretreatment with 24-Epi prior to MPP^+^ administration indicates low ROS levels, as revealed by rhodamine and MitoSOX Red fluorescence, suggesting that the relatively low SOD activity induced by 24-Epi pretreatment may have already scavenged MPP^+^-generated ROS before 24 h. 

CAT activity is another parameter of oxidative stress. Our results point out that at 15 h and 24 h, 24-Epi pretreatment reduces the MPP^+^-induced increase in CAT activity, confirming a scavenging role for 24-Epi in the pretreatment experimental condition, as already reported for another natural antioxidant molecule, sesamin [13]. GPx is a selenium-dependent enzyme involved in antioxidant defense and intracellular redox regulation and modulation. Cardiovascular and neuroprotective effects of the trace element selenium have been observed, although long-term supplementation has a “ying-yang” effect [80]. The glutathione response after MPP^+^ treatments has already been described in a DAergic cell line and is in accordance with our results, demonstrating an increase in GPx activity at 24 h after toxin administration [81]. Our data and other findings suggest a change in glutathione regulatory enzyme activities during the kinetics of MPP^+^ administration [81]. More interestingly, our data show a significant increase in GPx activity after 24-Epi pretreatment, indicating that this molecule may augment stock of the antioxidant enzyme above control levels.

In addition, our results demonstrate a clear neuroprotective and antiapoptotic role of 24-Epi against cellular death induced by MPP^+^ administration. We also document that 24-Epi is a potent modulator of apoptosis, opposing MPP^+^-induced DNA fragmentation and decreasing MPP^+^-evoked apoptotic/antiapoptotic protein expression, namely, the Bax/Bcl-2 ratio. Altogether, our data establish that oxidative stress-induced apoptosis in DAergic cells can be reversed by preadministration of 24-Epi. Thus, 24-Epi may be accounted for by another natural molecule interacting with intracellular apoptotic pathways [51, 82]. Recent studies have reported the anticancer and antiproliferative activities of 2 BRs, 28-homocastasterone and 24-Epi [35], supporting their cytotoxic and apoptotic role. This is not the first time that natural neuroprotective and antioxidant molecules appear to act as “double agents” on apoptotic parameters, depending on the cell lines studied and the concentrations tested. First, it should be noted that to demonstrate the anticancer and antiproliferative activities of BRs, these authors used micromolar concentrations, and toxicity was apparent at 10 *μ*M and higher dose levels [35]. In our study, we tested nanomolar concentrations of 24-Epi since micromolar levels would likely be difficult to sustain *in vivo* in the human brain. On the other hand, neuronal PC12 cells are differentiated cells expressing a neuronal phenotype as well as DAT, neurofilament proteins, and ER*α* and ER*β*, in contrast to native mitotic PC12 cells, where 17-*β* estradiol or several polyphenols do not counteract MPP^+^-induced cellular death [50, 55]. It is certainly important in future work to study *in vivo* models of PD to better understand the role of plant steroids in mammalian neuronal systems.

Finally, this is the first investigation to highlight 24-Epi's powerful function in parameters of neuronal cell distress, apoptosis, and cellular death. Other studies should be performed to elucidate the possible modulation of 24-Epi on the intrinsic parameters of DAergic neurotransmission. In addition, we cannot completely exclude a role of 24-Epi on MPP^+^ uptake, in particular via the modulation of DAT and VMAT expression, that could mimic downstream events. Though, altogether these results can open the way to further document, in an animal model of PD, the importance of BRs as natural molecules in preventive or/and complementary strategies to control neurodegeneration. 

## References

[1] Dauer W, Przedborski S (2003). Parkinson’ s disease: mechanisms and models. *Neuron*.

[2] Olanow CW (2007). The pathogenesis of cell death in Parkinson’s disease–2007. *Movement Disorders*.

[3] Zhou C, Huang Y, Przedborski S (2008). Oxidative stress in parkinson’ s disease: a mechanism of pathogenic and therapeutic significance. *Annals of the New York Academy of Sciences*.

[4] Keeney PM, Xie J, Capaldi RA (2006). Parkinson’ s disease brain mitochondrial complex i has oxidatively damaged subunits and is functionally impaired and misassembled. *The Journal of Neuroscience*.

[5] Navarro A, Boveris A, Bandez MJ (2009). Human brain cortex: mitochondrial oxidative damage and adaptive response in parkinson disease and in dementia with Lewy bodies. *Free Radical Biology and Medicine*.

[6] Calon F, Lavertu N, Lemieux AM (2001). Effect of MPTP-induced denervation on basal ganglia GABA receptors: correlation with dopamine concentrations and dopamine transporter. *Synapse*.

[7] Jakowec MW, Nixon K, Hogg E (2004). Tyrosine hydroxylase and dopamine transporter expression following 1-Methyl–4-Phenyl-1,2,3,6-tetrahydropyridine-induced neurodegeneration of the mouse nigrostriatal pathway. *Journal of Neuroscience Research*.

[8] Blanchet J, Longpre F, Bureau G (2008). Resveratrol, a red wine polyphenol, protects dopaminergic neurons in MPTP-treated mice. *Progress in Neuro-Psychopharmacology and Biological Psychiatry*.

[9] Mizuno Y, Sone N, Saitoh T (1987). Effects of 1-methyl–4-phenyl-1,2,3,6-tetrahydropyridine and 1-methyl–4-phenylpyridinium ion on activities of the enzymes in the electron transport system in mouse brain. *Journal of Neurochemistry*.

[10] Schober A (2004). Classic toxin-induced animal models of Parkinson’s disease: 6-OHDA and MPTP. *Cell and Tissue Research*.

[11] Przedborski S, Tieu K, Perier C, Vila M (2004). MPTP as a mitochondrial neurotoxic model of parkinson's disease. *Journal of Bioenergetics and Biomembranes*.

[12] Cassarino DS, Fall CP, Swerdlow RH (1997). Elevated reactive oxygen species and antioxidant enzyme activities in animal and cellular models of Parkinson’ s disease. *Biochimica Et Biophysica Acta*.

[13] Lahaie-Collins V, Bournival J, Plouffe M, Carange J, Martinoli MG (2008). Sesamin modulates tyrosine hydroxylase, superoxide dismutase, catalase, inducible NO synthase and interleukin-6 expression in dopaminergic cells under MPP-induced oxidative stress. *Oxidative Medicine and Cellular Longevity*.

[14] Thomas B, Saravanan KS, Mohanakumar KP (2008). In vitro and in vivo evidences that antioxidant action contributes to the neuroprotective effects of the neuronal nitric oxide synthase and monoamine oxidase-B inhibitor, 7-nitroindazole. *Neurochemistry International*.

[15] Ara J, Przedborski S, Naini AB (1998). Inactivation of tyrosine hydroxylase by nitration following exposure to peroxynitrite and 1-methyl–4-phenyl-1,2,3,6-tetrahydropyridine (MPTP). *Proceedings of the National Academy of Sciences of the United States of America*.

[16] Adams JD, Klaidman LK, Leung AC (1993). MPP^+^and MPDP^+^induced oxygen radical formation with mitochondrial enzymes. *Free Radical Biology and Medicine*.

[17] Shang T, Kotamraju S, Kalivendi SV, Hillard CJ, Kalyanaraman B (2004). 1-Methyl–4-phenylpyridinium-induced apoptosis in cerebellar granule neurons is mediated by transferrin receptor iron-dependent depletion of tetrahydrobiopterin and neuronal nitric-oxide synthase-derived superoxide. *Journal of Biological Chemistry*.

[18] Hartley A, Stone JM, Heron C, Cooper JM, Schapira AH (1994). Complex i inhibitors induce dose-dependent apoptosis in PC12 cells: relevance to parkinson’ s disease. *Journal of Neurochemistry*.

[19] Park KH, Yokota T, Sakurai A, Takahashi N (1987). Occurence of castasterone, brassinolide and methyl 4-chloroindole-3-acetate in immature Vicia faba seeds. *Agricultural and Biological Chemistry*.

[20] Ikekawa N, Nishiyama F, Fujimoto Y (1988). Identification of 24-epibrassinolide in bee pollen of the broad bean, Vicia faba L.. *Chemical and Pharmaceutical Pulletin*.

[21] Gamoh K, Omote K, Okamoto N, Takatsuto S (1989). High-performance liquid chromatography of brassinosteroids in plants with derivatization using 9-phenanthreneboronic acid. *Journal of Chromatography*.

[22] Khripach VA, Zhabinskii VN, de Groot AE (1999). *Brassinosteroids: A New Class of Plant Hormones*.

[23] Khripach V, Zhabinsk V, De Groot A (2000). Twenty years of brassinosteroids: steroidal plant hormones warrant better crops for the XXI century. *Annals of Botany*.

[24] Mazorra LM, Nùnez M, Hechavarria M, Coll F, Sànchez-Blanco MJ (2002). Influence of brassinosteroids on antioxidant enzymes activity in tomato under different temperatures. *Biologia Plantarum*.

[25] Ali B, Hayat S, Fariduddin Q, Ahmad A (2008). 24-Epibrassinolide protects against the stress generated by salinity and nickel in Brassica juncea. *Chemosphere*.

[26] Özdemir F, Bor M, Demiral T (2004). Effects of 24-epibrassinolide on seed germination, seedling growth, lipid peroxidation, proline content and antioxidative system of rice(Oryza sativa L.)under salinity stress. *Plant Growth Regulation*.

[27] Calabrese V, Butterfield DA, Stella AM (2003). Nutritional antioxidants and the heme oxygenase pathway of stress tolerance: novel targets for neuroprotection in Alzheimer’ s disease. *The Italian Journal of Biochemistry*.

[28] Anekonda TS (2006). Resveratrol-a boon for treating Alzheimer’ s disease?. *Brain Research Reviews*.

[29] Baur JA, Sinclair DA (2006). Therapeutic potential of resveratrol: the in vivo evidence. *Nature Reviews Drug Discovery*.

[30] Singh M, Arseneault M, Sanderson T (2008). Challenges for research on polyphenols from foods in Alzheimer’ s disease: bioavailability, metabolism, and cellular and molecular mechanisms. *Journal of Agricultural and Food Chemistry*.

[31] Alcain FJ, Villalba JM (2009). Sirtuin activators. *Expert Opinion on Therapeutic Patents*.

[32] Pandey KB, Rizvi SI (2009). Plant polyphenols as dietary antioxidants in human health and disease. *Oxidative Medicine and Cellular Longevity*.

[33] Baur JA (2010). Resveratrol, sirtuins, and the promise of a DR mimetic. *Mechanisms of Ageing and Development*.

[34] Iriti M, Vitalini S, Fico G, Faoro F (2010). Neuroprotective herbs and foods from different traditional medicines and diets. *Molecules*.

[35] Malikova J, Swaczynova J, Kolar Z, Strnad M (2008). Anticancer and antiproliferative activity of natural brassinosteroids. *Phytochemistry*.

[36] Wu YD, Lou YJ (2007). Brassinolide, a plant sterol from pollen of Brassica napus L., induces apoptosis in human prostate cancer PC-3 cells. *Pharmazie*.

[37] Slavikova B, Kohout L, Budesinsky M, Swaczynova J, Kasal A (2008). Brassinosteroids: synthesis and activity of some fluoro analogues. *Journal of Medicinal Chemistry*.

[38] Hamdy AH, Aboutabl EA, Sameer S (2009). 3-Keto-22-epi-28-nor-cathasterone, a brassinosteroid-related metabolite from Cystoseira myrica. *Steroids*.

[39] Ramirez JA, Teme Centurin OM, Gros EG, Galagovsky LR (2000). Synthesis and bioactivity evaluation of brassinosteroid analogs. *Steroids*.

[40] Michelini FM, Ramirez JA, Berra A, Galagovsky LR, Alché LE (2004). In vitro and in vivo antiherpetic activity of three new synthetic brassinosteroid analogues. *Steroids*.

[41] Wachsman MB, Ramirez JA, Talarico LB, Galagovsky LR, Coto CE (2004). Antiviral activity of natural and synthetic brassinosteroids. *Current Medicinal Chemistry*.

[42] Farina E, Piu P, Strinna L (1974). Extraction of L-DOPA from vicia faba L. and other plants of the leguminous genera. *Bollettino della Società italiana di biologia sperimentale*.

[43] Rabey JM, Vered Y, Shabtai H, Graff E, Harsat A, Korczyn AD (1993). Broad bean(Vicia faba)consumption and Parkinson’s disease. *Advances in Neurology*.

[44] LeWitt PA (2009). Levodopa therapeutics for Parkinson’s disease: new developments. *Parkinsonism and Related Disorders*.

[45] Apaydin H, Ertan S, Ozekmekci S (2000). Broad bean(Vicia faba)- a natural source of L-dopa-prolongs “on” periods in patients with Parkinson’ s disease who have “on-off” fluctuations. *Movement Disorders*.

[46] Raguthu L, Varanese S, Flancbaum L, Tayler E, Di Rocco A (2009). Fava beans and Parkinson's disease: useful ’natural supplement’ or useless risk?. *European Journal of Neurology*.

[47] Greene LA, Tischler AS (1976). Establishment of a noradrenergic clonal line of rat adrenal pheochromocytoma cells which respond to nerve growth factor. *Proceedings of the National Academy of Sciences of the United States of America*.

[48] Ryu EJ, Angelastro JM, Greene LA (2005). Analysis of gene expression changes in a cellular model of Parkinson disease. *Neurobiology of Disease*.

[49] Gelinas S, Martinoli MG (2002). Neuroprotective effect of estradiol and phytoestrogens on MPP+-induced cytotoxicity in neuronal PC12 cells. *Journal of Neuroscience Research*.

[50] Gagne B, Gelinas S, Bureau G (2003). Effects of estradiol, phytoestrogens, and Ginkgo biloba extracts against 1-methyl-4-phenyl-pyridine-induced oxidative stress. *Endocrine*.

[51] Bournival J, Quessy P, Martinoli MG (2009). Protective effects of resveratrol and quercetin against MPP+-induced oxidative stress act by modulating markers of apoptotic death in dopaminergic neurons. *Cellular and Molecular Neurobiology*.

[52] Korzeniewski C, Callewaert DM (1983). An enzyme-release assay for natural cytotoxicity. *Journal of Immunological Methods*.

[53] Martin A, Clynes M (1991). Acid phosphatase: endpoint for in vitro toxicity tests. *In Vitro Cellular and Developmental Biology*.

[54] Decker T, Lohmann-Matthes ML (1988). A quick and simple method for the quantitation of lactate dehydrogenase release in measurements of cellular cytotoxicity and tumor necrosis factor(TNF)activity. *Journal of Immunological Methods*.

[55] Gelinas S, Bureau G, Valastro B (2004). Alpha and beta estradiol protect neuronal but not native PC12 cells from paraquat-induced oxidative stress. *Neurotoxicity Research*.

[56] Henderson LM, Chappell JB (1993). Dihydrorhodamine 123: a fluorescent probe for superoxide generation?. *European Journal of Biochemistry*.

[57] Kooy NW, Royall JA, Ischiropoulos H (1994). Peroxynitrite-mediated oxidation of dihydrorhodamine 123. *Free Radical Biology and Medicine*.

[58] Wardman P (2007). Fluorescent and luminescent probes for measurement of oxidative and nitrosative species in cells and tissues: progress, pitfalls, and prospects. *Free Radical Biology and Medicine*.

[59] Wrona M, Patel K, Wardman P (2005). Reactivity of 2’ , 7’-dichlorodihydrofluorescein and dihydrorhodamine 123 and their oxidized forms toward carbonate, nitrogen dioxide, and hydroxyl radicals. *Free Radical Biology and Medicine*.

[60] Mates JM, Sanchez-Jimenez F (1999). Antioxidant enzymes and their implications in pathophysiologic processes. *Frontiers in Bioscience*.

[61] Klein JA, Ackerman SL (2003). Oxidative stress, cell cycle, and neurodegeneration. *Journal of Clinical Investigation*.

[62] Chowdhury I, Tharakan B, Bhat GK (2008). Caspases-an update. *Comparative Biochemistry and Physiology B*.

[63] Mignotte B, Vayssiere JL (1998). Mitochondria and apoptosis. *European Journal of Biochemistry*.

[64] Hartmann A, Hunot S, Michel PP (2000). Caspase-3: a vulnerability factor and final effector in apoptotic death of dopaminergic neurons in Parkinson’ s disease. *Proceedings of the National Academy of Sciences of the United States of America*.

[65] Wu Y, Shang Y, Sun SG (2007). Protective effect of erythropoietin against 1-methyl-4-phenylpyridinium- induced neurodegenaration in PC12 cells. *Neuroscience Bulletin*.

[66] Chiasson K, Lahaie-Collins V, Bournival J, Delapierre B, Gélinas S, Martinoli M-G (2006). Oxidative stress and 17-*α*- and 17-*β*-estradiol modulate neurofilaments differently. *Journal of Molecular Neuroscience*.

[67] Kadota T, Yamaai T, Saito Y (1996). Expression of dopamine transporter at the tips of growing neurites of PC12 cells. *Journal of Histochemistry and Cytochemistry*.

[68] Nilsen J, Mor G, Naftolin F (1998). Raloxifene induces neurite outgrowth in estrogen receptor positive PC12 cells. *Menopause*.

[69] Yasukazu Y, Etsuo N (2003). Antioxidant effects of phytosterol and its components. *Journal of Nutritional Science and Vitaminology*.

[70] Zhao HB, Wang SZ, He QIH (2005). Ganoderma total sterol(GS)and GS1 protect rat cerebral cortical neurons from hypoxia/reoxygenation injury. *Life Sciences*.

[71] Chen XC, Zhou YC, Chen Y, Zhu Y-G, Fang F, Chen L-M (2005). Ginsenoside Rg1 reduces MPTP-induced substantia nigra neuron loss by suppressing oxidative stress. *Acta Pharmacologica Sinica*.

[72] Vivancos M, Moreno JJ (2005). *β*-sitosterol modulates antioxidant enzyme response in RAW 264.7 macrophages. *Free Radical Biology and Medicine*.

[73] Chen XC, Zhu YG, Zhu LA (2003). Ginsenoside Rg1 attenuates dopamine-induced apoptosis in PC12 cells by suppressing oxidative stress. *European Journal of Pharmacology*.

[74] Chen XC, Zhu YG, Wang XZ, Zhu L-A, Huang C (2001). Protective effect of ginsenoside Rg1 on dopamine-induced apoptosis in PC12 cells. *Acta Pharmacologica Sinica*.

[75] Rosati F, Danza G, Guarna A (2003). New evidence of similarity between human and plant steroid metabolism: 5alpha-reductase activity in Solanum malacoxylon. *Endocrinology*.

[76] Fricke CB, Schroder M, Poulsen M (2007). Increased plant sterol and stanol levels in brain of Watanabe rabbits fed rapeseed oil derived plant sterol or stanol esters. * The British Journal of Nutrition*.

[77] An LJ, Guan S, Shi GF (2006). Protocatechuic acid from Alpinia oxyphylla against MPP+-induced neurotoxicity in PC12 cells. *Food and Chemical Toxicology*.

[78] Hou RC, Wu CC, Yang CH, Jeng K-CG (2004). Protective effects of sesamin and sesamolin on murine BV-2 microglia cell line under hypoxia. *Neuroscience Letters*.

[79] Jung TW, Lee JY, Shim WS (2006). Rosiglitazone protects human neuroblastoma SH-SY5Y cells against acetaldehyde-induced cytotoxicity. *Biochemical and Biophysical Research Communications*.

[80] Steinbrenner H, Sies H (2009). Protection against reactive oxygen species by selenoproteins. *Biochimica et Biophysica Acta*.

[81] Drechsel DA, Liang LP, Patel M (2007). 1-Methyl–4-phenylpyridinium-induced alterations of glutathione status in immortalized rat dopaminergic neurons. *Toxicology and Applied Pharmacology*.

[82] Bureau G, Longpre F, Martinoli MG (2008). Resveratrol and quercetin, two natural polyphenols, reduce apoptotic neuronal cell death induced by neuroinflammation. *Journal of Neuroscience Research*.

